# Supplementary material for: Heat adaptation of phage T7 under an extended genetic code
Source: Virus Evol. 2021 Dec 1;7(2):veab100. doi: 10.1093/ve/veab100 (PMC8923235; doi:10.1093/ve/veab100)
Supplement: veab100_Supp [file veab100_supp.zip › Sequencing_Analysis.zip!/Sequencing_Analysis/S3/evidence/RA_27.html]

BRESEQ :: Evidence


| Read alignment evidence... | | | | | | | | | | | |
| --- | --- | --- | --- | --- | --- | --- | --- | --- | --- | --- | --- |
|  | seq id | position | | ref | new | freq | score (cons/poly) | reads | annotation | genes | product |
| \* | NC\_001604.1 | 26,306 | 0 | A | G | 62.2% | 1388.1 / inf | 1812 | intergenic (–/–) | *–/–* | –/– |
| Reads supporting (aligned to +/- strand):  **ref** base A (330/345);  **new** base G (567/544);  **total** (915/897) | | | | | | | | | | | |
| Fisher's exact test for biased strand distribution *p*-value = 3.80e-01 | | | | | | | | | | | |
| Kolmogorov-Smirnov test that lower quality scores support variant *p*-value = 8.72e-01 | | | | | | | | | | | |

Only 100 of 1820 total aligned reads displayed.  

`ACCCTATAGACGTAGCTGTGAGTACCAACCGAATAGCAATCCTTAAGTACGCCGTTCCGTTCTCAGAAGAGTTACTCATCTGGTCCGATGAAGCACAATTCGTCCTGACTGCCTCGGGTACTCTCACATCTAAGTCGGTTGAGTTGAACCTAACGACCCAGTTTGACGTACAGGACCGAGCGAGACCTTTTGGGATTGGGCGTAATGTCTACTTTGCTAGTCCGAGGTCCAGCTTCACGTCCATCCACAGGTACTACGCTGTGCAGGATGTCAGTTCCGTTAAGAATGCTGAGGACATT‑ACATCACACGTTCCTAACTACATCCCTAATGGTGTGTTCAGTATTTGCGGAAGTGGTACGGAAAACTTCTGTTCGGTACTATCTCACGGGGACCCTAGTAAAATCTTCATGTACAAATTCCTGTACCTGAACGAAGAGTTAAGGCAACAGTCGTGGTCTCATTGGGACTTTGGGGAAAACGTACAGGTTCTAGCTTGTCAGAGTATCAGCTCAGATATGTATGTGATTCTTCGCAATGAGTTCAATACGTTCCTAGCTAGAATCTCTTTCACTAAGAACGCCATTGACTTACAGGGAGAAC  >  NC_001604.1/26007‑26606`  

                                                                                                                                                                                                                                                                                                            `|`                                                                                                                                                                                                                                                                                                              
`acCCTATAGACGTAGCTGTGAGTACCAACCGAATAGCAATCCTTAAGTACGCCGTTCCGTTCTCAGAAGAGTTACTCATCTGGTCCGATGAAGCACAATTCGTCCTGACTGCCTCGGGTACTCTCACATCTAAGTCGGTTGCGTTGAACCTAACGACCCAGTTTGACGTACAGGACCGAGCGAGACCTTTTGGGATTGGGCGTAATGTCTACTTTGCTAGTCCGAGGTCCAGCTTCACGTCCATCCACAGGTACTACGCTGTGCAGGATGTCAGTTCCGTTAAGAATGCTGAGGACATT‑Ac                                                                                                                                                                                                                                                                                                             >  1:293212/1‑301 (MQ=255)`  
   `cTATAGACGTAGCTGTGAGTACCAACCGAATAGCAATCCTTAAGTACGCCGTTCCGTTCTCAGAAGAGTTACTCATCTGGTCCGATGAAGCACAATTCGTCCTGACTGCCTCGGGTACTCTCACATCTAAGTCGGTTGAGTTGAACCTAACGACCCAGTTTGACGTACAGGACCGAGCGAGACCTTTTGGGATTGGGCGTAATGTCTACTTTGCTAGTCCGAGGTCCAGCTTCACGTCCATCCACAGGTACTACGCTGTGCAGGATGTCAGTTCCGTTAAGAATGCTGAGGCCATT‑GCATc                                                                                                                                                                                                                                                                                                          >  1:277717/1‑301 (MQ=255)`  
     `ataGCCGTAGCTGTGAGTACCAACCGAATAGCAATCCTTAAGTACACCGTTCCGTTCTCAGAAGAGTTACTCATCTGGTCCGATGAAGCACAATTCGTCCTGACTGCCTCGGGTACTCTCACATCTAAGTCGGTTGAGTTGAACCTAACGACCCAGTTTGACGTACAGGACCGAGCGAGACCTTTTGGGATTGGGCGTAATGTCTACTTTGCTAGTCCGAGGTCCAGCTTCACGTCCATCCACAGGTACTACGCTGTGCAGGATGTCAGTTCCGTTAAGAATGCTGAGGACGTT‑ACATcac                                                                                                                                                                                                                                                                                                        <  1:309686/301‑1 (MQ=255)`  
       `aGACGTAGCTGTGAGTACCAACCGAATAGCAATCCTTAAGTACGCCGTTCCGTTCTCAGAAGAGTTACTCATCTGGTCCGATGAAGCACAATTCGTCCTGACTGCCTCGGGTACTCTCACATCTAAGTCGGTTGCGTTGAACCTAACGACCCAGTTTGACGTACAGGACCGAGCGAGACCTTTTGGGATTGGGCGTAATGTCTACTTTGCTAGTCCGAGGTCCAGCTTCACGTCCATCCACAGGTACTACGCTGTGCAGGATGTCAGTTCCGTTAAGAATGCTGAGGACATT‑ACATCacac                                                                                                                                                                                                                                                                                                      <  1:282807/301‑1 (MQ=255)`  
       `aGACGTAGCTGTGAGTACCAACCGAATAGAAATCCTTAAGTACGCCGTTCCGTTCTCAGAAGAGTTACTCATCTGGTCCGATGAAGCACAATTCGTCCTGACTGCCTCGGGTACTCTCACATCTAAGTCGGTTGAGTTGAACCTAACGACCCAGTTTGACGTACAGGACCGAGCGAGACCTTTTGGGATTGGGCGTAATGTCTACTTTGCTAGTCCGAGGTACAGCTTCACGTCCATCCACAGGTACTACGCTGTGCAGGATGTCAGTTCCGTTAAGAATGCTGAGGACATG‑GCATaacaa                                                                                                                                                                                                                                                                                                      >  1:221381/1‑300 (MQ=255)`  
         `aCGTAGCTGTGAGTACCAACCGAATAGCAATCCTTAAGTACGCCGTTCCGTTCTCAGAAGAGTTACTCATCTGGTCCGATGAAGCACAATTCGTCCTGACTGCCTCGGGTACTCTCACATCTAAGTCGGTTGAGTTGAACCTAACGACCCAGTTTGACGTACAGGACCGAGCGAGACCTGTTGGGATTGGGCGTAATGTCTACTTTGCTAGGCCGCGGTCCAGCTTCACGTCCATCCACAGGTACTACGCTGTGCAGGAGGTCAGTTCCGTTAAGAAGGCTGGGGACATT‑ACATCACACgg                                                                                                                                                                                                                                                                                                    >  1:324391/1‑300 (MQ=255)`  
            `tAGCTGTGAGTACCAACCGAATAGCAATCCTTAAGTACGCCGTTCCGTTCTCAGAAGAGTTACTCATCTGGTCCGATGAAGCACAATTCGTCCTGACTGCCTCGGGTACTCTCACATCTAAGTCGGTTGAGTTGAACCTAACGACCCAGTTTGACGTACAGGACCGAGCGAGACCTTTTGGGATTGGGAGTAATGTCTACTTTGCTAGTCCGAGGTCCAGCTTCACGTCCATCCACAGGTACTACGCTGTGCAGGATGTCAGTTCCGTTAAGAATGCTGAGGACATT‑GCATCACACGTTcc                                                                                                                                                                                                                                                                                                 >  1:17383/1‑301 (MQ=255)`  
            `tAGCTGTGAGTACCAACCGAATAGCAATCCTTAAGTACGCCGTTCCGTTCTCAGAAGAGTTACTCATCTGGTCCGATGAAGCACAATTCGTCCTGACCTCCTCGGGTACTCTCACATCTAAGTCGGTTGAGTTGAACCTAACGACCCAGTTTGACGTACAGGACCGAGCGAGACCTTTTGGGATTGGGCGTAATGTCTACTTTGCTAGTCCGAGGTCCAGCTTCACGTCCATCCACAGGTACTACGCTGTGCAGGATGTCAGTTCCGTTAAGAATGCTGAGGAAATT‑GCATCACACGTTcc                                                                                                                                                                                                                                                                                                 <  1:74906/301‑1 (MQ=255)`  
                `tgtgAGTACCAACCGAATAGCAATCCTTAAGTACGCCGTTCCGTTCTCAGAAGAGTTACTCATCTGGTCCGATGAAGCACAATTCGTCCTGACTGCCTCGGGTACTCTCACATCTAAGTCGGTTGAGTTGAACCTAACGACCCAGTTTGACGTACAGGACCGAGCGAGACCTTTTGGGATTGGGCGTAATGTCTACTTTGCTAGTCCGAGGTCCAGCTTCACGTCCATCCACAGGTACTACGCTGTGCAGGATGTCAGTTCCGTTAAGAATGCTGAGGACATT‑ACATCACACGTTCCTAAc                                                                                                                                                                                                                                                                                             <  1:204197/301‑1 (MQ=255)`  
                `tgtgAGTACCAACCGAATAGCAATCCTTAAGTACGCCGTTCCATTCTCAGAAGAGTTACTCATCTGGTCCGATGAAGCACAATTCGTCCTGACTGCCTCGGGTACTCTCACATCTAAGTCGGTTGAGTTGAACCTAACGACCCAGTTTGACGTACAGGACCGAGCGAGACCTTTTGGGATTGGGCGTAATGTCTACTTTGCTAGTCCGAGGTCCAGCTTCACGTCCATCCACAGGTACTACGCTGTGCAGGATGTCAGTTCCGTTAAGAATGCTGAGGACATT‑GCATCACACGTTCCTAAc                                                                                                                                                                                                                                                                                             <  1:218263/301‑1 (MQ=255)`  
                 `gtgAGTACCAATCGAATAGCAATCCTTAAGTACGCCGTTCCGTTCTCAGAAGAGTTACTCATCTGGTCCGATGAAGCACAATTCGTCCTGACTGCCTCGGGTACTCTCACATCTAAGTCGGTTGCGTTGAACCTAACGACCCAGTTTGACGTACAGGACCGAGCGAGACCTTTTGGGATTGGGCGTAATGTCTACTTTGCTAGTCCGAGGTCCAGCTTCACGTCCATCCACAGGTACTACGCTGTGCAGGATGTCAGTTCCGTTAAGAATGCTGAGGACATT‑ACATCACACGTTCCTAACt                                                                                                                                                                                                                                                                                            <  1:124353/301‑1 (MQ=255)`  
                 `gggAGTACCAACCGAATAGCAATCCTTAAGTACGCCGTTCCGTTCTCAGAAGAGTTACTCATCTGGTCCGATGAAGCACAATTCGTCCTGACTGCCTCGGGTACTCTCACATCTAAGTCGGTTGAGTTGAACCTAACGACCCAGTTTGACGTACAGGACCGAGCGAGACCTTTTGGGATTGGGCGTAATGTCTACTGTGCTAGTCCGAGGTCCAGCTTCACGTACATCCACAGGTACTACGCTGTGCAGGATGTCAGTTCCGTTAAGAATGCTGAGGACATT‑GCATCACACGTTCCTAACt                                                                                                                                                                                                                                                                                            <  1:340690/299‑1 (MQ=255)`  
                 `gatgtgtataagagacaggCAATCCTTAAGTACGCCGTTCCGTTCTCAGAAGAGTTACTCATCTGGTCCGATGAAGTACAATTCGTCCTGACTGCCTCGGGTACTCTCACAACTAAGTCGGTTGAGTTGAACCTAACGACCCAGTTTGACGTACAGGACCGAGCGAGACCTTTTGGGATTGGGCGTAATGTCTACTTTGCTAGTCCGAGGTCCAGCTTCACGTCCATCCACAGGTACTACGCTGTGCAGGATGTCAGTTCCGTTAAGAATGCTGAGGACATT‑GCATCACACGTTCCTAACt                                                                                                                                                                                                                                                                                            <  1:313146/283‑1 (MQ=255)`  
                   `gAGTACCAACCGAATAGCAATCCTTAAGTACGCCGTTCCGTTCTCAGAAGAGTTACTCATCTGGTCCGATGAAGCACAATTCGTCCTGACTGCCTCGGGTACTCTCACATCTAAGTCGGTTGAGTTGAACCTAACGACCCAGTTTGACGTACAGGACCGAGCGAGACCTTTTGGGATTGGGCGTAATGTCTACTTTGCTAGTCCGAGGTCCAGCTTCACGTCCATCCACAGGTACTACGCTGTGCAGGATGTCAGTTCCGTTAAGAATGCTGAGGACATT‑GCATCACACGTTCCTAACTAc                                                                                                                                                                                                                                                                                          <  1:230866/301‑1 (MQ=255)`  
                   `gAGTACCAACCGAATAGCAATCCTTAAGTACACCGTTCCGTTCTCAGAAGAGTTACTCATCTGGTCCGATGAAGCACAATTCGTCCTGACTGCCTCGGGTACTCTCACATCTAAGTCGGTTGAGTTGAACCTAACGACCCAGTTTGACGTACAGGACCGAGCGAGACCTTTTGGGATTGGGCGTAATGTCTACTTTGCTAGTCCGAGGTCCAGCTTCACGTCCATCCACAGGTACTACGCTGTGCAGGATGTCAGTTCCGTTAAGAATGCTGAGGACATT‑ACATCACACGTTCCTAACTAc                                                                                                                                                                                                                                                                                          <  1:328272/301‑1 (MQ=255)`  
                           `aCCGAATAGCAATCCTTAAGTACGCCGTTCCGTTCTCAGAAGAGTTACTCATCTGGTCCGATGAAGCACAATTCGTCCTGACTGCCTCGGGTACTCTCACATCTAAGTCGGTTGAGTTGAACCTAACGACCCAGTTTGACGTACAAGACCGAGCGAGACCTTTTGGGATTGGGCGTAATGTCTACTTTGCTAGTCCGAGGTCCAGCTTCACGTCCATCCACAGGTACTACGCTGTGCAGGATGTCAGTTCCGTTAAGAATGCTGAGGACATT‑GCATCACACGTTCCTAACTACATCCCTaa                                                                                                                                                                                                                                                                                  <  1:197842/301‑1 (MQ=255)`  
                           `aCCGAATAGCAATCCTTAAGTACACCGTTCCGTTCTCAGAAGAGTTACTCATCTGGTCCGATGAAGCACAATTCGTCCTGACTGCCTCGGGTACTCTCACATCTAAGTCGGTTGAGTTGAACCTAACGACCCAGTTTGACGTACAGGACCGAGCGAGACCTTTTGGGATTGGGCGTAATGTCTACTTTGCTAGTCCGAGGTCCAGCTTCACGTCCATCCACAGGTACTACGCTGTGCAGGATGTCAGTTCCGTTAAGAATGCTGAGGACATT‑ACATCACACGTTCCTAACTACATCCCTaa                                                                                                                                                                                                                                                                                  >  1:247403/1‑301 (MQ=255)`  
                            `ccGAATAGCAATCCTTAAGTACGCCGTTCCGTTCTCAGAAGAGTTACTCATCTGGTCCGATGAAGCACAATTCGTCCTGACTGCCTCGGTTACTCTCACATCTAAGTCGGTTGAGTTGAACCTAACGACCCAGTTTGACGTACAGGACCGAGCGAGACCTTTTGGGATTGGGCGTAATGTCTACTTTGCTCGTCCGAGATCCAGCTTCTCGTCCATCTACAGGGACTACGCTGTGCAGGATGTCATTTCCGTTACGAATGTTGAGGACTTT‑GGCTCCCACGTTCCTAACTACTTCCCTAAt                                                                                                                                                                                                                                                                                 >  1:200534/1‑301 (MQ=255)`  
                              `gAATAGCAATCCTTAAGTACGCCGTTCCGTTCTCAGAAGAGTTACTCATCTGGTCCGATGAAGCACAATTCGTCCTGACTGCCTCGGGTACTCTCACATCTAAGTCGGTTGAGTTGAACCTAACGACCCAGTTTGACGTACAGGACCGAGCGAGACCTTTTGGGATTGGGCGTAATGTCTACTTTGCTAGTCCGAGGTCCAGCTTCACGTCCATCCACAGGTACTACGCTGTGCAGGATGTCAGTTCCGTTAAGAATGCTGAGGACATT‑GCATCACACGTTCCTAACTACATCCCTAATgg                                                                                                                                                                                                                                                                               <  1:139996/301‑1 (MQ=255)`  
                              `gAATAGCAATCCTTAAGTACACCGTTCCGTTCTCAGAAGAGTTACTCATCTGGTCCGATGAAGCACAATTCGTCCTGACTGCCTCGGGTACTCTCACATCTAAGTCGGTTGAGTTGAACCTAACGACCCAGTTTGACGTACAGGACCGAGCGAGACCTTTTGGGATTGGGCGTAATGTCTACTTTGCTAGTCCGAGGTCCAGCTTCACGTCCATCCACAGGTACTACGCTGTGCAGGATGTCAGTTCCGTTAAGAATGCTGAGGACATT‑ACATCACACGTTCCTAACTACATCCCTAATgg                                                                                                                                                                                                                                                                               >  1:184193/1‑301 (MQ=255)`  
                              `gAATAGCAATCCTTAAGGACACCGTTCCGTTCTCAGAAGAGTTACTCATCTGGTCCGATGAAGCACAATTCGTCCTGACTGCCTCGGGTACTCTCACATCTAAGTCGGTTGAGTTGTATCTAACGACCCAGTTTGACGTACAGGACCGAGCGAGACCTTTTGGGATTGGGCGTAATGTCTACTTTGCTAGTCCGAGGTCCAGCTTCACGTCCATCCACAGGTACTACGCTGTGCAGGATGTCAGTTCCGTTAAGAATGCTGAGGACATT‑ACATCACACGTTCCTAACTACATCCCTAATgg                                                                                                                                                                                                                                                                               <  1:90464/301‑1 (MQ=255)`  
                              `aaaTAGCAACTCTGAAGTACGCCCGTCCGTTCTCATAAGAGTCACTCAACTCGTCCGGCGAAGCATTATTCGTCCTGACTGCCTCGGGTACTCTCACATCTAAGTCGGTTGAGTTGAACCTAACGACCCAGTTTGACGTACAGGACCGAGCGACACCTTTTGGGATTGGGCGTAATGTCTACTTTGCTAGTCCGAGGTCCAGCTTCACGTCCATCCACAGGGACTACGCTGTGCAGGATGTCAGTTCCGTTAAGAATGCTGAGGACATT‑GCATCACACGTTCCTAACTACATCCCTAATgg                                                                                                                                                                                                                                                                               <  1:121131/300‑1 (MQ=255)`  
                                `aTAGAAATCCGTAAGTACGCAGTTCCGTTCTCAGAAGAGGTATGAATCGGGTCAGATGAAGCACAATTCGTCCTGACTGCATCGGGTACTATAACATCTAAGTAGGTTGAGTTGAACCTAACGACCCAGTTTGACGAACAGGACCGAGCGAGACCTTTTGGGATTGGGCGTAATGTCTACTTTGCTAGTCCGAGGGCCAGCTTCACGTCCATCCACAGGTACTACGCTGTGCAGGATGTCAGTTCCGTTAAGAATGCTGAGGACATT‑ACATCACACGTTCCTAACTACATCCCTAATGgtg                                                                                                                                                                                                                                                                             <  1:29642/301‑1 (MQ=255)`  
                                   `gCAATCCTTAAGTACGCCGTTCCGTTCTCAGAAGAGTTACTCATCTGGTCCGATGAAGCACAATTCGTCCTGACTGCCTCGGGTACTCTCACATCTAAGTCGGTTGCGTTGAACCTAACGACCCAGTTTGACGTACAGGACCGAGCGAGACCTTTTGGGATTGGGCGTAATGTCTACTTTGCTAGTCCGAGGTCCAGCTTCACGTCCATCCACAGGTACTACGCTGTGCAGGATGTCAGTTCCGTTAAGAATGCTGAGGACATT‑ACATCACACGTTCCTAACTACATCCCTAATGgtgtgt                                                                                                                                                                                                                                                                          <  1:97335/301‑1 (MQ=255)`  
                                      `aTCCTTAAGTACGCCGTTCCGTTCTCAGAAGAGTTACTCATCTGGTCCGATGAAGCACAATTCGTCCTGACTGCCTCGGGTACTCTCACATCTAAGTCGGTTGAGTTGAACCTAACGACCCAGTTTGACGTACAGGACCGAGCGAGACCTTTTGGGATTGGGCGTAATGTCTACTTTGCTAGTCCGAGGTCCAGCTTCACGTCCATCCACAGGTACTACGCTGTGCAGGATGTCAGTTCCGTTAAGAATGCTGAGGCCATT‑GCATCACACGTTCCTAACTACATCCCTAATggctgtctct                                                                                                                                                                                                                                                                       >  1:344905/1‑293 (MQ=255)`  
                                      `aTCCTTAAGTACGCCGTTCCGTTCTCAGAAGAGTTACTCATCTGGTCCGATGAAGCACAATTCGTCCTGACTGCCTCGGGTACTCTCACATCTAAGTCGGTTGAGTTGAACCTAACGACCCAGTTTGACGTACAGGACCGAGCGAGACCTTTTGGGATTGGGCGTAATGTCTACTTTGCTAGTCCGAGGTCCAGCTTCACGTCCATCCACAGGTACTACGCTGTGCAGGATGTCAGTTCCGTTAAGAATGCTGAGGACATT‑GCATCACACGTTCCTAAcctgtctcttatacacatctccc                                                                                                                                                                                                                                                                       >  1:16054/1‑279 (MQ=255)`  
                                      `aTCCTTAAGTACGCCGTTCCGTTCTCAGAAGAGTTACTCATCTGGTCCGATGAAGCACAATTCGTCCTGACTGCCTCGGGTACTCTCACATCTAAGTCGGTTGAGTTGAACCTAACGACCCAGTTTGACGTACAGGACCGAGCGAGACCTTTTGGGATTGGGCGTAATGTCTACTTTGCTAGTCCGAGGTCCAGCTTCACGTCCATCCACAGGTACTACGCTGTGCAGGATGTCAGTTCCGTTAAGAATGCTGAGGACATT‑GCATCACACGTTCCTAACTACATCCCTAATGGTGTGTTCa                                                                                                                                                                                                                                                                       >  1:227126/1‑301 (MQ=255)`  
                                      `aTCCTTAAGTACGCCGTTCCGTTCTCAGAAGAGTTACTCATCTGGTCCGATGAAGCACAATTCGTCCTGACTGCCTCGCGTACTCTCACATCTAAGTCGGTTGAGTTGAACCTAACGACCCAGTTTGACGTACAGGACCGAGCGAGACCTTTTGGGATTGGGCGTAATGTCTACTTTGCTAGTCCGAGGTCCAGCTTCACGTCCATCCACAGGTACTACGCTGTGCAGGATGTCCGTTCCGTTATGAATGCTGCGGCCATT‑TCATCACACGTTCCTAAATACATCCCTAATGGTGTGTTCa                                                                                                                                                                                                                                                                       >  1:83813/1‑301 (MQ=255)`  
                                      `aTCCTTAAGTACACCGTTCCGTTCTCAGAAGAGTTACTCATCTGGTCCGATGAAGCACAATTCGTCCTGACTGCCTCGGGTACTCTCACATCTAAGTCGGTTGAGTTGAACCTAACGACCCAGTTTGACGTACAGGACCGAGCGAGACCTTTTGGGATTGGGCGTAATGTCTACTTTGCTAGTCCGAGGTCCAGCTTCACGTCCATCCACAGGTACTACGCTGTGCAGGATGTCAGTTCCGTTAAGAATGCTGAGGACATT‑ACATCACACGTTCCTAACTACATCCCTAATGgtgctgtct                                                                                                                                                                                                                                                                       >  1:244822/1‑295 (MQ=255)`  
                                               `ctcgaagatgtgtataagagacagTTACTCATCTGGCCCGAGGAAGCACAATTCGTCCTGACTGCCTCGGGTACTCTCACATCTAAGTCGGTTTAGTTGAACCTAACGACCCAGTTTGACGTACAGGACCGAGCGAGACCTTTTGGGATTGGGCGTAATGTCTACTTTGCTAGTCCGAGGTCCAGCTTCACGTCCATCCACAGGTACTACGCTGTGCAGGATGTCAGTTCCGTTAAGAATGCTGAGGACATT‑GCATCACACGTTCCTAACTACATCCCTAATGGTGTGTTCAGTATTTGCg                                                                                                                                                                                                                                                              <  1:27537/279‑1 (MQ=255)`  
                                                `ccacccttccgttcTCAGAAGAGTTACTCATCTGGTCCGATGAAGCGCAATTCGTCCTGACTGCCTCGGTTACTCTCACATCTAAGTCGGTTGAGTTGAACCTAACGACCCAGTTTGACGTACAGGACCGAGCGAGACCTTTTGGGATTGGGCGTAATGTCTACTTTGCTAGTCCGAGGTCCAGCTTCACGTCCATCCACAGGTACTACGCTGTGCAGGATGTCAGTTCCGTTAAGAATGCTGAGGACATT‑ACATCACACGTTCCTAACTACATCCCTAATGGTGTGTTCAGTATTTGCgg                                                                                                                                                                                                                                                             <  1:46769/295‑1 (MQ=255)`  
                                                   `ccgttccgttCTCAGAAGAGTTACTCATCTGGTCCGATGAAGCACAATTCGTCCTGACTGCCTCGGGTACTCTCACATCTAAGTCGGTTGAGTTGAACCTAACGACCCAGTTTGACGTACAGGACCGAGCGAGACCTTTTGGGATTGGGCGTAATGTCTACTTTGCTAGTCCGAGGTCCAGCTTCACGTCCATCCACAGGTACTACGCTGTGCAGGATGTCAGTTCCGTTAAGAATGCTGAGGACATT‑GCATCACACGTTCCTAACTACATCCCTAATGGTGTGTTCAGTATTTGCGGAAg                                                                                                                                                                                                                                                          <  1:43508/301‑1 (MQ=255)`  
                                                                      `gTTACTCATCTGGTCCGATGAAGCACAATTCGTCCTGACTGCCTCGGGTACTCTCACATCTAAGTCGGTTGAGTTGAACCTAACGACCCAGTTTGACGTACAGGACCGAGCGAGACCTTTTGGGATTGGGCGTAATGTCTACTTTGCTAGTCCGAGGTCCAGCTTCACGTCCATCCACAGGTACTACGCTGTGCAGGATGTCAGTTCCGTTTAGAATGCTGAGGACATT‑GCATCACACGTTCCTAACTACATCCCTAATGGTGTGTTCAGTATTTGCGGAAGTGGTACGGAACACTTCTgc                                                                                                                                                                                                                                       >  1:189504/1‑300 (MQ=255)`  
                                                                      `gTTACTCATCTGGTCCGATGAAGCACAATTCGTCCTGACTGCCTCGGGTACTCTCACATCTAAGTCGGTTGAGTTGAACCTAACGACCCAGTTTGACGTACAGGACCGAGCGAGACCTTTTGGGATTGGGCGTAATGTCTACTTTGCTAGTCCGAGGTCCAGCTTCACGTCCATCCACAGGTACTACGCTGTGCAGGATGTCAGTTCCGTTAAGAATGCTGAGGACATT‑GCATCACACGTTCCTAACTACATCCCTAATGGTGTGTTCAGTATTTGCGGAAGTGGTACGGAAAACTTCTGt                                                                                                                                                                                                                                       >  1:304191/1‑301 (MQ=255)`  
                                                                                 `ggTCCGATGAAGCACAATTCGTCCTGACTGCCTCGGGTACTCTCACATCTAAGTCGGTTGAGTTGAACCTAACGACCCAGTTTGACGTACAGGACCGAGCGAGACCTTTTGGGATTGGTCGTAATGTCTACTTTGCTCGTCCGAGGTCCAGCTTCACGTCCATCCACAGGTACTACGCTGTGCAGGATGTCAGTTCCGTTAAGAATGCTGAGGACATT‑GCATCACACGTTCCTAACTACATCCCTAATGGTGTGTTCAGTATTTGCGGAAGTGGTACGGAAAACTTCTGTTCGGTACTAtt                                                                                                                                                                                                                            >  1:145506/1‑300 (MQ=255)`  
                                                                                 `ggTCCGATGAAGCACAATTCGTCCTGACTGCCTCGGGTACTCTCACATCTAAGTCGGTTGAGTTGAACCTAACGACCCAGTTTGACGTACAGGACCGAGCGAGACCTTTTGGGATTGGGCGTAATGTCTACTTTGCTAGTCCGAGGTCCAGCTTCACGTCCATCCACAGGTACTACGCTGTGCAGGATGTCAGTTCCGTTAAGAATGCTGAGGACATT‑GCATCACACGTTCCTAACTACATCCCTAATGGTGTGTTCCGTATTTGCGGAAGTGGTACGGAAAACTTCTGTTCGGTACTAtc                                                                                                                                                                                                                            >  1:286331/1‑301 (MQ=255)`  
                                                                                  `gTCCGATGAAGCACAATTCGTCCTGTCTGCCTCGTGTACTCTCACATCTAAGTCGGTTGAGTTGAACCTAACGCCCCAGTTTGACGTACAGGACCGAGCGAGACCTTTTGGGATTGGGCGTAATGTCTACTTTGCTAGTCCGAGGTCCAGCTTCACGTCCATCCACAGGTACTACGCTGTGCAGGATGTCAGTTCCGTTAAGAATGCTGAGGACATT‑ACATCACACGTTCCTAACTACATCCCTAATGGTGTGTTCAGTATTTGCGGAAGTGGTACGGAAAACTTCTGTTCGGTACTAtct                                                                                                                                                                                                                           <  1:127803/301‑1 (MQ=255)`  
                                                                                      `gATGAAGCACAATTCGTCCTGACTGCCTCGGGTACTCTCACATCTAAGTCGGTTGAGTTGAACCTAACGACCCAGTTTGACGTACAGGACCGAGCGAGACCTTTTGGGATTGGGCGTAATGTCTACTTTGCTAGTCCGAGGTCCAGCTTCACGTCCATCCACAGGTACTACGCTGTGCAGGATGTCAGTTCCGTTAAGAATGCTGAGGACATT‑GCATCACACGTTCCTAACTACATCCCTAATGGTGTGTTCAGTATTTGCGGAAGTGGTACGGAAAACTTCTGTTCGGTACTATCTCACg                                                                                                                                                                                                                       <  1:239097/301‑1 (MQ=255)`  
                                                                                       `aTGAAGCACAATTCGTCCTGACTGCCTCGGGTACTCTCACATCTAAGTCGGTTGAGTTGAACCTAACGACCCAGTTTGACGTACAGGACCGAGCGAGACCTTTTGGGATTGGGCGTAATGTCTACTTTGCTAGTCCGAGGTCCAGCTTCACGTCCATCCACAGGTACTACGCTGTGCAGGATGTCAGTACCGTTAAGAATGCTGAGGACATT‑ACATCACACGTTCCTAACTACATCCCTAATGGTGTGTTCAGTATTTGCGGAAGTGGTACGAAAAACTTCTGTTCGGTACTATCGCACgg                                                                                                                                                                                                                      >  1:39049/1‑301 (MQ=255)`  
                                                                                            `gCACAATTCGTCCTGACTGCCTCGGGTACTCTCACATCTAAGTCGGTTGAGTTGAACCTAACGACCCAGTTTGACGTACAGGACCGAGCGAGACCTTTTGGGATTGGGCGTAATGTCTACTTTGCTAGTCCGAGGTCCAGCTTCACGTCCATCCACAGGTACTACGCTGTGCAGGATGTCAGTTCCGTTAAGAATGCTGAGGACATT‑ACATCACACGTTCCTAACTACATCCCTAATGGTGTGTTCAGTATTTGCGGAAGTGGTACGGAAAACTTCTGTTCGGTTCTATCTCACGGGGAcc                                                                                                                                                                                                                 >  1:224427/1‑301 (MQ=255)`  
                                                                                                `aaTTCGTCCTGACTGCCTCGGGTACTCTCACATCTAAGTCGGTTGGGTTGAACCTAACGACCCAGTTTGACGTACAGGACCGAGCGAGACCTTTTGGGATTGGGCGTAATGTCTACTTTGCTAGTCCGAGGTCCAGCTTCAAGTCCATCCACTGGTACTACGCTGTGCAGGATGTCAGTTCCGTTAAGAATGCTGAGGACATT‑GCATCACACGTTACTAACTACATCCCTAATGGTGTGTTCAGTATTTGCGAGAGTGGTACGGAAAACTTCTGTTCGGTACTATCTCACGGGGACCCTAg                                                                                                                                                                                                             >  1:16832/1‑301 (MQ=255)`  
                                                                                                   `tCGTCCTGACTGACTCGGGTACTCTCACATCTAAGTCGGTTGAGTTGAACCTAACGACCCAGTTTGACGTACAGGACCGAGCGAGACCTTTTGGGATTGGGCGTAATGTCTACTTTGCTAGTCCGAGGTCCAGCTTCACGTCCATCCACAGGTACTACGCTGTGCAGGATGTCAGTTCCGTTAAGAATGCTGAGGACATT‑GCATCACACGTTCCTAACTACATCCCTAATGGTGTGTTCAGTATTTGCGGAAGTGGTACGGAAAACTTCTGTTCGGTACTATCTCACGGGGACCCTAGTaa                                                                                                                                                                                                          <  1:114431/301‑1 (MQ=255)`  
                                                                                                   `ataagagacaggCCTCGGGTACGTTCACATCTAAGTCGGTTGAGTTGAACCTAACGACCCAGTTTGACGTACAGGACCGAGCGAGACCTTTTGGGATTGGGCGTAATGTCTACTTTGCTAGTCCGAGGTCCAGCTTCATGTCCATCCAGAGGTACTACGCTGTGCAGGATGTCAGTTCCGTTAAGAATGCTGAGGACATT‑GAATCACACGTTCCTAACTACATCCCTAATGGTGTGTTCAGTATTTGCGGAAGTGGTACGGAAAACTTCTGTTCGGTACTATCTCGCGGGGACCCTAGTaa                                                                                                                                                                                                          <  1:17845/290‑1 (MQ=255)`  
                                                                                                                             `acaTCTAAGTCGGTTGGGTTGAACCTAACGACCCAGTTTGACGTACAGGACCGAGCGAGACCTTTTGGGATTGGGCGTAATGTCTACTTTGCTAGTCCGAGGTCCAGCTTCACGTCCATCCACAGGTACTACGCTGTGCAGGATGTCAGTTTCGTTAAGAATGCTGAGGACATT‑GCATCACACGTTCCTAACTACATCCCTAATGGTGTGTTCAGTATTTGCGGAAGTGGTACGGAAAACTTCTGTTCGGTACTATCTCACGGGGACCCTAGTAAAATCTTCATGTACAAATTCCTGTAcc                                                                                                                                                                                <  1:30134/301‑1 (MQ=255)`  
                                                                                                                                  `tAAGTCGGTTGCGTTGAACCTAACGACCCAGTTTGACGTACAGGACCGAGCGAGACCTTTTGGGATTGGGCGTAATGTCTACTTTGCTAGTCCGAGGTCCAGCTTCACGTCCATCCACAGGTACTACGCTGTGCAGGGTGTCAGTTCCGTTAAGAATGCTGAGGACATT‑GCATCACACGTTCCTAACTACATCCCTAATGGTGTGTTCAGTATTTGCGGAAGTGGTACGGAAAACTTCTGTTCGGTACTATCTCACGGGGACCCTAGTAAAATCTTCATGTACAAATTCCTGTACCTGAAc                                                                                                                                                                           >  1:142684/1‑301 (MQ=255)`  
                                                                                                                                     `gTCGGTTGAGTTGAACCTAACGACCCAGTTTGACGTACAGGACCGAGCGAGACCTTTTGGGATTGGGCGTAATGTCTACTTTGCTAGTCCGAGGTCCAGCTTCACGTCCATCCACAGGTACTACGCTGTGCAGGATGTCAGTTCCGTTAAGAATGCTGAGGACATT‑GCATCACACGTTCCTAACTACATCCCTAATGGTGTGTTCAGTATTTGCGGAAGTGGTACGGAAAACTTCTGTTCGGTACTATCTCACGGGGACCCTAGTAAAATCTTCATGTACAAATTCCTGTACCTGAAcctt                                                                                                                                                                        >  1:289730/1‑298 (MQ=255)`  
                                                                                                                                         `gttgcgttgaACCTAACGACCCAGTTTGACGTACAGGACCGAGCGAGACCTTTTGGGATTGGGCGTAATGTCTACTTTGCTAGTCCGAGGTCCAGCTTCACGTCCATCCACAGGTACTACGCTGTGCAGGATGTCAGTTCCGTTAAGAATGCTGAGGACATT‑GCATCACACGTTCCTAACTACATCCCTAATGGTGTGTTCAGTATTTGCGGAAGTGGTACGGAAAACTTCTGTTCGGTACTATCTCACGGGGACCCTAGTAAAATCTTCATGTACAAATTCCTGTACCTGAACGATGAGt                                                                                                                                                                    >  1:20915/1‑301 (MQ=255)`  
                                                                                                                                         `gttgagttgaACCTAACGACCCAGTTTGACGTACAGGACCGAGCGAGACCTTTTGGGATTGGGCGTAATGTCTACTTTGCTAGTCCGAGGTCCAGCTTCACGTCCATCCACAGGTACTACGCTGTGCAGGATGTCAGTTCCGTTAAGAATGCTGAGGACATT‑ACATCACACGTTCCTAACTACATCCCTAATGGTGTGTTCAGTATTTGCGGAAGTGGTACGGAAAACTTCTTTTCGGTACTATCTCACGGGGACCCTAGTAAACTCTTCATGTACAAATTCCTGTACCTGAACGAAGAGt                                                                                                                                                                    >  1:298740/1‑301 (MQ=255)`  
                                                                                                                                             `agttgaACCTAACGACCCAGTTTGACGTACAGGACCGAGCGAGACCTTTTGGGCTTGGGCGTAATGTCTACTTTGCTAGTCCGAGGTCCAGCTTCACGTCCATCCACAGGTACTACGCTGTGCAGGATGTCAGTTCCGTTAAGAATGCTGAGGACATT‑GCATCACACGTTCCTAACTACATCCCTAATGGTGTGTTCAGTATTTGCGGAAGTGGTACGGAAAACTTCTGTTCGGTAATATCTCACGGGGACCCTAGTAAAATCTCCATGTACAAATTCCTGTACCTGAACGAAGAGTTaaa                                                                                                                                                                >  1:72094/1‑300 (MQ=255)`  
                                                                                                                                              `gttgaACCTAACGACCCAGTTTGACGTACAGGACCGAGCGAGACCTTTTGGGATTGGGCGTAATGTCTACTTTGCTAGTCCGAGGTCCAGCTTCCCGTCCATCCACAGGTACTACGCTGTGCACGATGTCAGTTCCGTTAAGAATGCTGAGGACATT‑GCATCACACGTTCCTAACTACATCCCTAATGGTGTGTTCAGTATTTGCGGAAGTGGTACGGAAAACTTCTGTTCGGTAATATCTCACGGGCACCCTAGTAACATCTTCATGTACACATTACTGTACCTGAACGAAGAGTTAAgg                                                                                                                                                               >  1:242276/1‑301 (MQ=255)`  
                                                                                                                                              `gttgaACCTAACGACCCAGTTTGACGTACAGGACCGAGCGAGACCTTTTGGGATTGGGCGTAATGTCTACTTTGCTAGTCCGAGGTCCAGCTTCACGTCCATCCACAGGTACTACGCTGTGCAGGATGTCAGTTCCGTTAAGAATGCTGAGGACATT‑GCATCACACGTTCCTAACTACATCCCTAATGGTGTGTTCAGTATTTGCGGAAGTGGTACGGAAAACTTCTGTTCGGTACTATCTCACGGGGACCCTAGTAAAATCTTCATGTACAAATTCCTGTACCTGAACGAAGAGTTAAgg                                                                                                                                                               <  1:107122/301‑1 (MQ=255)`  
                                                                                                                                                `tgaACCTAACGACCCAGTTTGACGTACAGGACCGAGCGAGACCTTTTGGGATTGGGCGTAATGTCTACTTTGCTAGTCCGAGGTCCAGCTTCACGTCCATCCACAGGTACTACGCTGTGCAGGATGTCAGTTCCGTTAAGAATGCTGAGGACATT‑GCATCACATGTTCCTAACTACATCCCTAATGGTGTGTTCAGTATTTGCGGAAGTGGTACGGAAAACTTCTGTTCGGTACTATCTCACGGGGACCCTAGTAAAATCTTCATGTACAAATTCCTGTACCTGAACGAAGAGTTAAGGCa                                                                                                                                                             <  1:148924/301‑1 (MQ=255)`  
                                                                                                                                                `tgaACCAAACGACCAAGTTTGACGTACAGGACCGAGCGAGACCTTTTGGGATTGGGCGTAATGTCTACTTTGCTAGTCCGAGGTCCAGCTTCACGTCCATCCACAGGTACTACGCTGTGCAGGATGTCAGTTCCGTTAAGAATGCTGAGGACATT‑GCATCACACGTTCCTAACTACATCCCTAATGGTGTGTTCAGTATTTGCGGAAGTGGTACGGAAAACTTCTGTTCGGTACTATCTCACGGGGACCCTAGTAAAATCTTCATGTACAAATTCCTGTACCTGAACGAAGAGTTAAGGCa                                                                                                                                                             <  1:100095/301‑1 (MQ=255)`  
                                                                                                                                                           `aCCCAGTTTGACGTACAGGACCGAGCGAGACCTTTTGGGATTGGGCGTAATGTCTACTTTGCTAGTCCGAGGTCCAGCTTCACGTCCATCCACAGGTACTACGCTGTGCAGGATGTCAGTTCCGTTAAGAATGCTGAGGACATT‑GCATCACACGTTCCTAACTACATCCCTAATGGTGTGTTCAGTATTTGCGGAAGTGGTACGGAAAACTTCTGTTCGGTACTATCTCACGGGGACCCTAGTAAAATCTTCATGTACAAATTCCTGTACCTGAACGAAGAGTTAAGGCAACAGTCGTGGt                                                                                                                                                  >  1:53227/1‑301 (MQ=255)`  
                                                                                                                                                            `cccAGTATGACGTACAGGACCGAGCGAGACCTTTTGGGATTGGGCGTAATGTCTACTTTGCTAGTCCGAGGTCCAGCTTCACGTCCATCCACAGGTACTACGCTGTGCAGGATGTCAGTTCCGTTAAGAATGCTGAGGACATT‑GCATCACACGTTCCTAACTACATCCCTAATGGTGTGTTCAGTATTTGCGGAAGTGGTACGGAAAACTTCTGTTCGGTACTATCTCACGGGGACCCTAGTAAAATCTTCATGTACAAATTCCTGTACCTGAACGAAGAGTTAAGGCAACAGTCGTGGtc                                                                                                                                                 <  1:255427/301‑1 (MQ=255)`  
                                                                                                                                                                `gTTTGACGTACAGGACCGAGCGAGACCTTTTGGGATCGGGCGTAATGTCTACTTTGCTAGTCCTAGGTCCAGCTTCACGTCCATCAACA‑GTACTACGCTGTGCCGGATGTCAGTTCCGTTAAGAATGATGAGGAAATT‑ACATCACACGTTCCTAACAACATCCCTAAAGGTGTGTACAGTATTTGCGGAAGAGGTACGGAAAACTACTGTTAGGTACTATCTCACGGGGACCATAGTAAAATCTTCAAGTACACATTCCTGTACCTGAACGAAGAGATTAGGCCActgctgacttttatac                                                                                                                                            >  1:272299/1‑286 (MQ=255)`  
                                                                                                                                                                            `ggACCGAGCGAGACCTTTTGGGATTGGGCGTAATGTCTACTTTGCTAGTCCGAGGTCCAGCTTCACGTCCATCCACAGGTACTACGCTGTGCAGGATGTCAGTTCCGTTAAGAATGCTGAGGACATT‑GCATCACACGTTCCTAACTACATCCCTAATGGTGTGTTCAGTATTTGCGGAAGTGGTACGGAAAACTTCTGTTCGGTACTATCTCACGGGGACCCTAGTAAAATCTTAATGTACAAATTCCTGTACCTGAACGAAGAGTTACGGCAACAGTCGTGGTCTCATTGGGACTTTggg                                                                                                                                 >  1:111501/1‑301 (MQ=255)`  
                                                                                                                                                                                         `ccTTTTGGGATTGGGCGTAATGTCTACTTTGCTAGTCCGAGGTCCAGCTTCACGTCCATCCACAGGTACTACGCTGTGCAGGATGTCAGTTCCGTTAAGAATGCTGAGGACATT‑ACATCACACGTTCCTAACTACATCCCTAATGGTGTGTTCAGTATTTGCGGAAGTGGTACGGAAAACTTCTGTTCGGTACTATCTCACGGGGACCCTAGTAAAATCTTCATGTACAAATTCCTGTACCTGAACGAAGAGTTAAGGCAACAGTCGTGGTCTCATTGGGACTTTGGGGAAAACGTACAgg                                                                                                                    >  1:333784/1‑301 (MQ=255)`  
                                                                                                                                                                                          `cTTTTGGGATTGGGCGTAATGTCTACTTTGCTAGTCCGAGGTCCAGCTTCACGTCCATCCACAGGTACTACGCTGTGCAGGATGTCAGTTCCGTTAAGAATGCTGAGGACATT‑GCATCACACGTTCCTAACTACATCCCTAATGGTGTGTTCAGTATTTGCGGAAGTGGTACGGAAAACTTCTGTTCGGTACTATCTCACGGGGACCCTAGTAAAATCTTCATGTACAAATTCCTGTACCTGAACGAAGAGTTAAGGCAACAGTCGTGGTCTCATTGGGACTTTGGGGAAAACGTACAGGt                                                                                                                   >  1:234141/1‑301 (MQ=255)`  
                                                                                                                                                                                          `cTTTTGGGATTGGGCGTAATGTCTACTTTGCTAGTCCGAGGTCCAGCTTCACGTCCATCCACAGGTACTACGCTGTGCAGGATGTCAGATCCGTTAAGAATGCCGAGGACATT‑ATATCACACGTTCCTAACTACATCCCTAATGGTGTGTTCAGTATTTGCGGAAGTGGTACGGAAAACTTCTGTTCGGTACTATCTCACGGGGACCCTAGTAAAATCTTCATGTACAAATTCCTGTACCTGAACGAAGAGTTAAGGCAACAGTCGTGGTCTCATTGGGACTTTGGGGAAAAAGAACAggg                                                                                                                   >  1:306137/1‑300 (MQ=255)`  
                                                                                                                                                                                                `ggATTAGGCGTAATGTCTACTTTGCTAGTCCGAGGTCCAGCTTCACGTCCATCCACAGGTACTACGCTGTGCAGGATGTCAGTTCCGTTAAGAATGCTGAGGACATT‑ACATCACACGTTCCTAACTACATCCCTAATGGTGTGTTCAGTATTTGCGGAAGTGGTACGGAAAACTTCTGTTCGGTACTATCTCACGGGGACCCTAGTAAAATCTTCATGTACAAATTCCTGTACCTGAACGAAGAGTTAAGGCAACAGTCGTGGTCTCATTGGGACTTTGGGGAAAACGTACAGGTTCTAGc                                                                                                             <  1:63919/301‑1 (MQ=255)`  
                                                                                                                                                                                                 `gATTGGGCGTAATTTCTACTTTGCTAGTCCGAGGTCCAGCTTCACGTCCATCCACATGTACTACGCTGTGCAGGATGTCAGTTCCGTTAAGAATGCTTAGGACATT‑GCATCACACGTTCCTAACTACATCCCTAATGGTGTGTTCAGTATTTGCGGAAGTGGTACGGAAAACTTCTGTTCGGTACTATCTCACGGGGACCATAGTAAAATCTTCATGTACAAATTCCTGTACCTGAACGAAGAGTTAAGGCAACAGTCGTGTTCTCATTGGGACTTTGGGGAAAACGTACAGGTTCTAGCt                                                                                                            <  1:93776/301‑1 (MQ=255)`  
                                                                                                                                                                                                      `ggCGTAATGTCTACTTTGCTAGTCCGAGGTCCAGCTTCACGTCCATCCACAGGTACTACGCTGTGCAGGATGTCAGTTCCGTTAAGAATGCTGAGGACATT‑GCATCACACGTTCCTAACTACATCCCTAATGGTGTGTTCAGTATTTGCGGAAGTTGTACGGAAAGCTTCTGTTCGGTACTATCTCACGGGGACCCTAGTAAAATCTTCATGTACAAATTCCTGTACCTGAACGAAGAGTTAAGGCAACAGTCGTGGTCTCATTGGGACTTTGGGGACAACGTACAGGTTCTAGCTTGTcc                                                                                                       >  1:152247/1‑300 (MQ=255)`  
                                                                                                                                                                                                           `aaTGTCTACTTTGCTAGTCCGAGGTCCAGCTTCACGTCCATCCACAGGTACTACGCTGTGCAGGATGTCAGTTCCGTTAAGAATGCTGAGGACATT‑GCATCACACGTTCCTAACTACATCCCTAATGGTGTGTTCAGTATTTGCGGAAGTGGTACGGAAAACTTCTGTTCGGTACTATCTCACGGGGACCCTAGTAAAATCTTCATGTACAAATTCCTGTACCTGAACGAAGAGTTAAGGCAACAGTCGTGGTCTCATTGGGACTTTGGGGAAAACGTACAGGTTCTAGCTTGTCAGAGTa                                                                                                  <  1:266669/301‑1 (MQ=255)`  
                                                                                                                                                                                                             `tGTCTACTTTGCTAGTCCGAGGTCCAGCTTCACGTCCATCCACAGGTACTACGCTGTGCAGGATGTCAGTTCCGTTAAGAATGCTGAGGACATT‑GCATCACACGTTCCTAACTACATCCCTAATGGTGTGTTCAGTATTTGCGGAAGTGGTACGGAAAACTTCTGTTCGGTACTATCTCACGGGGACCCTAGTAAAATCCTCATGTACAAATTCCTGTACCTGAACGAAGAGTTAAGGCAACAGTCGTGGTCTCATTGGGACTTTGGGGAAAACGTACAGGTTCTAGCTTGTCAGAGTATc                                                                                                >  1:81013/1‑301 (MQ=255)`  
                                                                                                                                                                                                                     `ttGCTAGTCCGAGGTCCAGCTTCACGTCCATCCACAGGTACTACGCTGTGCAGGATGTCAGTTCCGTTAAGAATGCTGAGGACATT‑GCATCACACGTTCCTAACTACATCCCTAATGGTGTGTTCAGTATTTGCGGAAGTGGTACGGAAAACTTCTGTTCGGTACTATCTCACGGGGACCCTAGTAAAATCTTCATGTACAAATTCCTGTACCTGAACGAAGAGTTAAGGCAACAGTCGTGGTCTCATTGGGACTTTGGGGAAAACGTACAGGTTCTAGCTTGTCAGAGTATCAGCTCAGa                                                                                        >  1:77372/1‑301 (MQ=255)`  
                                                                                                                                                                                                                       `gCTAGTCCGAGTTCCAGCTTCACGTCCATCCACAGGTACTACGCTGTGCAGGATGTCAGTTCCGTTAAGAATGCTGAGGACATT‑GCATCACACGTTCCTAACTACATCCCTAATGGTGTGATCAGTATTTGCGGAAGTGGTACGGAAAACTTCTGTTCGGTACTATCTCACGGGGACCCTAGTAAAATCTTCATGTACAAATTCCTGTACCTGAACGAAGAGTTAAGGCAACAGTCGTGGTCTCATTGGGACTTTGGGGAAAACGTACAGGTTCTAGCTTGTCAGAGTATCAGCTCAGata                                                                                      <  1:269265/301‑1 (MQ=255)`  
                                                                                                                                                                                                                           `ttataagagacAGCTTCACGTCCATCCACAGGTACTACGCTGTGCAGGATGTCAGTTCCGTTAAGAATGCTGAGGACATT‑ACATCACACGTTCCTAACTACATCCCTAATGGTGTGTTCAGTATTTGCGGAAGTGGTACGGAAAACTTCTGTACGGTACTATCTCACGGGGACCCTAGTAAAATCTTCATGTACAAATTCCTGTACCTGAACGAAGAGTTAAGGCAACAGTCGTGGTCTCATTGGGACTTTGGGGAAAACGTACAGGTTCTAGCTTGTCAGAGTATCAGCTCAGAtatgta                                                                                  <  1:131402/291‑1 (MQ=255)`  
                                                                                                                                                                                                                             `ccGAGGTCCAGCTTCACGTCCATCCACAGGTACTACGCTGTGCAGGATGTCAGTTCCGTTAAGAATGCTGAGGACATT‑GCATCACACGTTCCTAACTACATCCCTAATGGTGTGTTCAGTATTTGCGGAAGTGGTACGGAAAACTTCTGTTCGGTACTATCTCACGGGGACCCTAGTAAAATCTTCATGTACAAATTCCTGTACCTGAACGAAGAGTTAAGGCAACAGTCGTGGTCTCATTGGGACTTTGGGGAAAACGTACAGGTTCTAGCTTGTCAGAGTATCAGCTCAGAtatgtatg                                                                                <  1:134908/301‑1 (MQ=255)`  
                                                                                                                                                                                                                                   `tCCAGCTTCACGTCCATCCACAGGTACTACGCTGTGCAGGATGTCAGTTCCGTTAAGAATTCTGAGGACATT‑ACATCACACGTTCCTAACTACATCCCTAATGGTGTGTTCAGTATTTGCGGAAGTGGTACGGAAAACTTCTGTTCGGTACTATCTCACGGGGACCCTAGTAAAATCTTCATGTACAAATTCCTGTACCTGAACGAAGAGTTAAGGCAACAGTCGTGGTCTCATTGGGACTTTGGGGAAAACGTACAGGTTCTAGCTTGTCAGAGTATCAGCTCAGATATGTATGTGAttc                                                                          <  1:326777/301‑1 (MQ=255)`  
                                                                                                                                                                                                                                   `tCCAGCTTCACGTCCATCCACAGGTACTACGCTGTGCAGGATGTCAGTTCCGTTAAGAATGCTGAGGACATT‑GCATCACACGTTCCTAACTACATCCCTAATGGTGTGTTCAGTATTTGCGGAAGTGGTACGGAAAACTTCTGTTCGGTACTATCTCACGGGGACCCTAGTAAAATCTTCATGTACAAATTCCTGTACCTGAACGAAGAGTTAAGGCAACAGTCGTGGTCTCATTGGGACTTTGGGGAAAACGTACAGGTTCTAGCTTGTCAGAGTATCAGCTCAGATATGTATGTGAttc                                                                          <  1:87278/301‑1 (MQ=255)`  
                                                                                                                                                                                                                                       `cgcctacatgggtgctcaGGTACTACGCTGTGCAGGATGTCAGTTCCGTTAAGAATGCTGAGGACATT‑GCATCACACGTTCCTAACTACATCCCTAATGGTGTGTTCAGTATTTGCGGAAGTGGTACGGAAAACTTCTGTTCGGTACTATCTCACGGGGACCCTAGTAAAATCTTCATGTACAAATTCCTGTACCTGAACGAAGAGTTAAGGCAACAGTCGTGGTCTCATTGGGACTTTGGGGAAAACGTACAGGTTCTAGCTTGTCAGAGTATCAGCTCAGATATGTATGTGATTCTTCg                                                                      >  1:207153/17‑301 (MQ=255)`  
                                                                                                                                                                                                                                            `acttccagccaCAGTTACTACGCTGTGCAGGGTGTCTGTTCCGTTAAGAATGCTGAGGACATT‑ACATCACACGTTCCTCAATACATCCCTAATGGTGTGTTCTGTATTTGCGGAAGTGGTACGGAAAACTTCTGTTCGGTACTATCTCACGGGGACCCTAGTAAAATCTTCATGTACAAATTCCTGTTCCTGAACGAAGAGTTAAGGCCACAGTCGTGGGCTCATTGGTACTTTGGGGAAAACGTACAGGTTTTAGCTTGTCAGAGTAACAGCTCAGATATGTATGTGATTCTTCGCAATg                                                                 <  1:102667/298‑1 (MQ=255)`  
                                                                                                                                                                                                                                                 `catccaCAGGTACTACGCTGTGCAGGATGTCAGTTCTGTTAAGAATGCTGAGGACATT‑ACATCACACGTTCCTAACTACATCCCTAATGGTGTGTTCAGTATTTGCGGAAGTGGTACGGAAAACTTCTGTTCGGTACTATCTCACGGGGACCCTAGTAAAATCTTCATGTACAAATTCCTGTACCTGAACGAAGAGTTAAGGCAACAGTCGTGGTCTCATTGGGACTTTGGGGAAAACGTACAGGTTCTAGCTTGTCAGAGTATCAGCTAAGCTATGTATGTGATTCTTCGCAATGAGttt                                                            >  1:118639/1‑300 (MQ=255)`  
                                                                                                                                                                                                                                                    `ctcggagatgggtataagagacaggggcAGTTCCGTTAAGAATGGTGAGGACATT‑GCATCACACGTTCCTAACTACATCCCTAATGGTGTGTTCAGTATTTTCGGAAGTGGTACGGAAAACTTCTGATCGGTACTATCTCACGGGGACCCTAGTAAAATCTTCATGTACAAATTCCTGTACCTGAACGAAGAGTTAAGGCAACAGTCGTGGTCTCATTGGGACTTTGGGGAAAACGTACAGGTTCTAGCTTGTCAGAGTATCAGCTCAGATATGTATGTGATTCTTCGCAATGAGTTCAAt                                                         <  1:316614/274‑1 (MQ=255)`  
                                                                                                                                                                                                                                                    `ccaCAGGTACTACGCTGTGCAGGATGACAGTTCCGTTAAGAATGCTGAGGACATT‑GCATCACACGTTCCTAACTACATCCCTAATGGTGTGTTCAGTATTTGCGGAAGTGGTACGGAAAACTTCTGTTCGGTACTATCTCACGGGGACCCTAGTAAAATCTTCATGTACAAATTCCTGTACCTGAACGAAGAGTTAAGGCAACAGTCGTGGTCTCATTGGGACTTGGTGGAAAACGTACAGGTTCTAGCTTGTCAGAGTATCAGCTCAGATATGTATGTGATTCTTCGCAATGAGTTCAAt                                                         <  1:195236/301‑1 (MQ=255)`  
                                                                                                                                                                                                                                                     `cacaGGTACTACGCTGTGCAGGATGTCAGTTCCGTTAAGAATGCTGAGGACATT‑GCATCACACGTTCCTAACTACATCCCTAATGGTGTGTTCAGTATTTGCGGAAGTGGTACGGAAAACTTCTGTTCGGTACTATCTCACGGGGACCCTAGTAAAATCTTCATGTACAAATTCCTGTACCTGAACGAAGAGTTAAGGCAACAGTCGTGGTCTCATTGGGACTTTGGGGAAAACGTACAGGTTCTAGCTTGTCAGAGTATCAGCTCAGATATGTATGTGATTCTTCGCAATGAGTTCAATa                                                        >  1:26360/1‑301 (MQ=255)`  
                                                                                                                                                                                                                                                      `tgtataagagacaggtgCAGGATGTCAGTTCCGTTAAGAATGCTGAGGACATT‑GCATCACACGTTCCTAACTACATCCCTAATGGTGTGTTCAGTATTTGCGGAAGTGGTACGGAAAACTTCTGTTCGGTACTATCTCACGGGGACCCTAGTAAAATCTTCATGTACAAATTCCTGTACCTGAACGAAGAGTTAAGGCAACAGTCGTGGTCTCATTGGGACTTTGGGGAAAACGTACAGGTTCTAGCTTGTCAGAGTATCAGCTCAGATATGTATGTGATTCTTCGCAATGAGTTCAATAc                                                       <  1:171582/287‑1 (MQ=255)`  
                                                                                                                                                                                                                                                      `acaGGTACTACGCTGTGCAGGATGTCAGTTCCGTTAAGAATGCTGAGGACATT‑ACATCACACGTTCCTAACTACATCCCTAATGGTGTGTTCAGTATTTGCGGAAGTGGTACGGAAAACTTCTGTTCGGTACTATCTCACGGGGACCCTAGTAAAATCTTCATGTACAAATTCCTGTACCTGAACGAATAGTTAAGGCAACAGTCGTGGTCTCATTGGGACTTTGGGGAAAACGTACAGGTTCTAGCTTGTCAGAGTATCAGCTCAGATATGTATGTGATTCTTCGCAATGAGTTCAATAc                                                       <  1:261503/301‑1 (MQ=255)`  
                                                                                                                                                                                                                                                      `aagggTACTGCGCTGTGCAGGATGTCAGTTCCGTTAAGAATGCTGAGGACATT‑ACATCACACGTTCCTAACTACATCCCTAATGGTGTGTTCAGTAGTTGCGGAAGTGGTACGGAAAACTTCTGTTCGGTACTATCTCACGGGGACCCTATTAAAATCTTCATGTACAAATTCCTGTACCTGAACGAAAAGTTAAGGCAACAGTCGTGGTCTCATTGGGACTTTGGGGAAAACGTACAGGTTCTAGCTTGTCAGAGTATCAGCTCAGATATGTATGTGATTCTTCGCAATGAGTTCAATAc                                                       <  1:320015/298‑1 (MQ=255)`  
                                                                                                                                                                                                                                                       `caGGTACTACGCTGTGCAGGATGTCAGTTCCGTTAAGAATGCTGAGGACATT‑GCATCACACGTTCCTAACTACATCCCTAATGGTGTGTTCAGTATTTGCGGAAGTGGTACGGAAAACTTCTGTTCGGTACTATCTCACGGGGACCCTAGTAAAATCTTCATGTACAAATTCCTGTACCTGAACGAAGAGTTAAGGCAACAGTCGTGGTCTCATTGGGACTTTGGGGGAAACGTACAGGTTCTAGCTTGTCAGAGTATCAGCTCAGATATGTATGTGATTCTTCGCAATGAGTTCAATACg                                                      >  1:60442/1‑301 (MQ=255)`  
                                                                                                                                                                                                                                                       `agggTACTACTCTGTGCAGGATGTCAGTTCCGTTAAGAATGCTGAGGACATT‑GCATCACACGTTCCTAACTACATCCCTAATGGTGTGTTCAGTATTTGCGGAAGTGGTACGGAAAACTTCTGTTCGGTACTATCTCACGGGGACCCTAGTAAAATCTTCATGTACAAATTCCTGTACCTGAACGAAGAGTTAAGGCAACAGTCGTGGTCTCATTGGGACTTTGGGGAAAACGTACAGGTTCTAGCTTGTCAGAGTATCAGCTCAGATATGTATGTGATTCTTCGCAATGAGTTCAATACg                                                      <  1:337174/299‑1 (MQ=255)`  
                                                                                                                                                                                                                                                           `tactacGCTGTGCAGGATGTCAGTTCCGTTAAGAATGCTGAGGACATTGACA‑CACACGTTCCTAACTACATCCCTAATGGTGTGTTCAGTATTTGCGGAAGTGGTACGGAAAACTTCTGTTCGGTACTATCTCACGGGGACCCTAGTAAAATCTTCATGTACAAATTCCTGTACCTGAACGAAGAGTTAAGGCAACAGTCGTGGTCTCATTGGGACTTTGGGGAAAACGTACAGGTTCTAGCTTGTCAGAGTATCAGCTCAGATATGTATGTGATTCTTCGCAATGAGTTCAATACGTTcc                                                  <  1:191875/301‑1 (MQ=255)`  
                                                                                                                                                                                                                                                               `acGCTGTGCAGGATGTCAGTTCCGTTAAGAATGCTGAGGACATT‑GCATCACACGTTCCTAACTACATCCCTAATGGTGTGTTCAGTATTTGCGGAAGTGGTACGGAAAACTTCTGTTCGGTACTATCTCACGGGGACCCTAGTAAAATCTTCATGTACAAATTCCTGTACCTGAACGAAGAGTTAAGGCAACAGTCGTGGTCTCATTGGGACTTTGGGGAAAACGTACAGGTTCTAGCTTGTCAGAGTATCAGCTCAGATATGTATGTGATTCTTCGCAATGAGTTCAATACGTTCctagc                                              >  1:250334/1‑301 (MQ=255)`  
                                                                                                                                                                                                                                                                `cGCTGTGCAGGATGTCAGTTCCGTTAAGAATGCTTAGGACATT‑ACATCACACGTTCCTAACTACATCCCTAATGGTGTGTTCAGTATTTGCGGAAGTGGTACGGAAAACTTCTGTTCGGTACTATCTCACGGGGACCCTAGTAAAATCTTCATGTACAAATTCCTGTACCTGAACGAAGAGTTAAGGCAACAGTCGTGGTCTCATTGGGACTTTGGGGAAAACGTACAGGTTCTAGCTTGTCAGAGTATCAGCTCAGATATGTATGTGATTCTTCGCAATGAGTTCAATACGTTCctagct                                             <  1:55544/301‑1 (MQ=255)`  
                                                                                                                                                                                                                                                                      `gCAGGATGTCAGTTCCGGTAAGAATGCTGAGGACATT‑GCATCACACGTTCCTAACTACATCCCTAATGGTGTGTTCAGTATTTGCGGAAGTGGTACGGAAAACTTCTGTTCGGTACTATCTCACGGGGACCCTAGTAAAATCTTCATGTACAAATTCCTGTACCGGAACGAAGAGTTAAGGCAACAGTCGTGGTATCATTGGGACTTTGGGGAAAACGTACAGGTTCTAGCTTGTAAGAGTATCAGCTCAGATATGTATGTGATTCTTCGCAATGAGTTCAATACGTTCCTAGCTAGAAtc                                       <  1:185988/301‑1 (MQ=255)`  
                                                                                                                                                                                                                                                                       `cAGGATGTCAGTTCCGTTAAGAATGCTGAGGACATT‑GCATCACACGTTCCTAACTACATCCCTAATGGTGTGGTCAGTATTTGCGGAAGTGGTACGGAAAACTTCTGTTCGGTACTATCTCACGGGGACCCTAGTAAAATCTTCATGTACAAATTCCTGTACCTGAACGAAGAGTTAAGGCAACAGTCGTGGTCTCATTGGGACTTTGGGGAAAACGTACAGGTTCTAGCTTGTCAGAGTATCAGCTCAGATATGTATGTGATTCTTCGCAATGAGTTCAATACGTTCCTAGCTAGAAtct                                      <  1:211786/301‑1 (MQ=255)`  
                                                                                                                                                                                                                                                                              `aggggtataagagacaggCTGAGGACATT‑GCATCACACGTTCCTAACTACATCCCTAATGGTGTGTTCAGTATTTGCGGAAGTGGTACGGAAAACTTCTGTTCGGTACTATCTAACGGGGACCCTAGTAAAATCTTCATGTACAAATTCCTGTACCTGAACGAAGAGTTAAGGCAACAGTCGTGGTCTCATTGGGACTTTGGGGAAAACGTACAGGTTCTAGCTTGTCAGAGTATCAGCTCAGATATGTATGTGATTCTTCGCAATGAGTTCAATACGTTCCTAGCTAGAATCTCTTTCAc                               <  1:105903/284‑1 (MQ=255)`  
                                                                                                                                                                                                                                                                                `cgTTCCGTTAAGAAATGTGAGGAAATT‑GCGTCACACGTTCCTAACTACATCCCTAATGGTGTGTTCAGTATTTGCGGAAGTGGTACGGAAAACTTCTGTTCGGTACTATCTCACGGGGACCCTAGTAAAATCTTCATGTACAAATTCCTGTACCTGAACGAAGAGTTAAGGCAACAGTCGTGGTCTCATTGTGACTTTGGGGAAAACGTACAGGTTCTAGCTTGTCAGAGTATCAGCTCAGATATGTATGTGATTCTTCGCAATGAGTTCAATACGTTCCTAGCTAGAATCTCTTTCACTa                             <  1:4943/300‑1 (MQ=255)`  
                                                                                                                                                                                                                                                                                    `cctttAAGAAGTCTTAGGATATT‑GTAGCACACGTTCCTAACTACATCCCTAATGGTGTGTTCAGTATGTGCGGAATGGGTACGGAAAACTTTTGTTCGGTACTAACTCACGTGGACCCTAGTAAAATCTTCATGTACAAATTCCTGTACCTGAACGAAGAGTTAAGGCAACAGTCGTGGTCTCATTTGGACTTTGGGGAAAACGTACAGGTTCTAGCTTGTCAGAGTATCAGCTCAGATATGTATGTGATTCTTCGCAATGAGTTCAATACGTTCCTAGCTAGAATCTCTTTCACTAAGaa                         <  1:66766/298‑1 (MQ=255)`  
                                                                                                                                                                                                                                                                                       `acaggAAGGCTGAGGACATT‑GCATCACACGTTCCTAAGTACATCCCTAAGGGTGTGTTCAGTATTTGCGGAAGTGGTACGGAAAACTTCTGTTCGGTACTATCTCACGGGGACCCTAGTAAAATCTTCATGTACAAATTCCTGTACCTGAACGAAGAGTTAAGGCAACAGTCGTGGTCTCATTGGGACTTTGGGGAAAACGTACAGGTTCTAGCTTGTCAGAGTATCAGCTCAGATATGTATGTGATTCTTCGCAATGAGTTCAATACGTTCCTAGCTAGAATCTCTTTCACTAAGAACGc                      <  1:236484/297‑1 (MQ=255)`  
                                                                                                                                                                                                                                                                                           `gAATGCTGAGGACATT‑GCATCACACGTTCCTAACTACATCCCTAATGGTGTGTTCAGTATTTGCGGAAGTGGTACGGAAAACTTCTGTTCGGTACTATCTCACGGGGACCCTAGTAAAATCTTCATGTACAAATTCCTGTACCTGAACGAAGAGTTAAGGCAACAGTCGTGGTCTCATTGGGACTTTGGGGAAAACGTACAGGTTCTAGCTTGTCAGAGTATCAGCTCAGATATGTATGTGATTCTTCGCAATGAGTTCAATACGTTCCTAGCTAGAATCTCTTTCACTAAGAACGCCAtt                  >  1:137343/1‑301 (MQ=255)`  
                                                                                                                                                                                                                                                                                              `tGCTGAGGACATT‑GCATCACACGTTCCTAACTACATCCCTAATGGTGTGTTCAGTATTTGCGGAAGTGGTACGGAAAACTTCTGTTCGGTACTATCTCACGGGGACCCTAGTAAAATCTTCATGTACAAATTCCTGTACCTGAACGAAGAGTTAAGGCAACAGTCGTGGTCTCATTGGGACTTTGGGGAAAACGTACAGGTTCTAGCTTGTCAGAGTATCAGCTCAGATATGTATGTGATTCTTCGCAATGAGTTCAATACGTTCCTAGCTAGAATCTCTTTCACTAAGAACGCCATTGAc               <  1:3476/301‑1 (MQ=255)`  
                                                                                                                                                                                                                                                                                               `gCTGAGGACATT‑GCATCACACGTTCCTAACTACATCCCTAATGGTGTGTTCAGTATTTGCGGAAGTGGTACGGAAAACTTCTGTTCGGTACTATCTCACGGGGACCCTAGTAAAATCTTCATGTACAAATTCCTGTACCTGAACGAAGAGTTAAGGCACCAGTCGTGGTCTCATTGGGACTTTGGGGAAAACGTACAGGTTCTAGCTTGTCAGAGTATCAGCTCAGATATGTCTGTGATTCTTCGCAATGAGTTCAATACGTTCCTAGCTAGAATCTCTTTCACTATGAACGCCATTGACt              >  1:164552/1‑301 (MQ=255)`  
                                                                                                                                                                                                                                                                                               `gCTGAGGACATT‑ACATCACACGTTCCTAACTACATCCCTAATGGTGTGTTCAGTATTTGCGGAAGTGGTACGGAAAACTTCTGTTCGGTACTATCTCACGGGGACCCTAGTAAAATCTTCATGTACAAATTCCTGTACCTGAACGAAGAGTTAAGGCAACAGTCGTGGTCTCATTGGGACTTTGGGGAAAACGTACAGGTTCTAGCTTGTCAGAGTATCAGCTCAGATATGTATGTGATTCTTCGCAATGAGTTCAATACGTTCCTAGCTAGAATCTCTTTCACTAAGAACGCTATTGACt              >  1:253149/1‑301 (MQ=255)`  
                                                                                                                                                                                                                                                                                                    `ggACATT‑GCATCACACGTTCCTAACTACATCCCTAATGGTGTGTTCAGTATTTGCGGAAGTGGTACGGAAAACTGCTGTTCGGTACTATCTCACGGGGACCCTAGTAAAATCTTCATGTACAAATTCCGGTACCTGAACGAAGAGTTAAGGCAACAGTCGTGGTCTCATTGGGACTTTGGGGAAAACGTACAGGTTCTAGCTTGTCAGAGTATCAGCTCAGATATGTATGTGATTCTTCGCAATGAGTTCAATACGTTCCTAGCTAGAATCTCTTTCACTAAGAACGCCATTGACTTACAg         <  1:216048/301‑1 (MQ=255)`  
                                                                                                                                                                                                                                                                                                     `gACATT‑GCATCACACGTTCCTAATTACATCCCTAATGGTGTGTTCAGTATTTGCGGAAGTGGTACGGAAAACTTCTGTTCGGTACTATCTCACGGGGACTCTAGTAAAATCTTCATGTACAAATTCCTGTACCTGAACGAAGAGTTAAGGCAACAGTCGTGGTCTCATTGGGACTTTGGGGAAAACGTACAGGTTCTAGCTTGTCAGAGTATCAGCTCAGATATGTATGTGATTCTTCGCAATGAGTTCAATACGTTCCTTGCTAGAATCTCTTTTACTAAGAACGCCATTGTCTTACAgg        >  1:258568/1‑301 (MQ=255)`  
                                                                                                                                                                                                                                                                                                       `cATT‑ACATCACACGTTCCTAACTACATCCCTAATGGTGTGTTCAGTATTTGCGGAAGTGGTACGGAAAACTTCTGTTCGGTACTATCTCACGGGGACCCTAGTAAAATCTTCATGTACAAATTCCTGTACCTGAACGAAGAGTTAAGGCAACAGTCGTGGTCTCATTGGGACTTTGGGGAAAACGTACAGGTTCTAGCTTGTCAGAGTATCAGCTCAGATATGTATGTGATTCTTCGCAATGAGTTCAATACGTTCCTAGCTAGAATCTCTTTCACTAAGAACGCCATTGACTTACAgctg      >  1:5789/1‑298 (MQ=255)`  
                                                                                                                                                                                                                                                                                                        `aTT‑GCATCACACTTTCCTAAGTACATCCCTAAAGTAGTGTTCAGTATTTGCTGAAGTGGTACGGAAAACTTCTGTTCGGTACTATCTCACGGGGAACCTAGTAAAATCTTCATGTACAAATTCCTGGACCTGAACGAAGAGTTAAGGCAACAGTAGTGGTCTCATTGGGACTTTGGGGAAAACGTACAGGTTCTAGCTTGTCAGAGTATCAGCTCAGATATGTATGTGATTCTTCGCAATGAGTTCAATACGTTCCTAGCTAGAATCTCTTTCACTAAGAACGCCATTGACTTACAGGgag     <  1:155853/301‑1 (MQ=255)`  
                                                                                                                                                                                                                                                                                                            `aCATCACACGTTCCTAACTACATCCCTAATGGTGTGTTCAGTATTTGCGGAAGTGGTACGGAAAACTTCTGTTCGGTACTATCTCACGGGGACCCTAGTAAAATCTTCATGTACAAATTCCTGTACCTGAACGAAGAGTTAAGGCAACAGTCGTGGTCTCATTGGGACTTTGGGGAAAACGTACAGGTTCTAGCTTGTCAGAGTATCAGCTCAGATATGTATGTGATTCTTCGCAATGAGTTCAATACGTTCCTAGCTAGAATCTCTTTCACTAAGAACGCCATTGACTTACAgctgtctc  >  1:40798/1‑294 (MQ=255)`  

                                                                                                                                                                                                                                                                                                            `|`                                                                                                                                                                                                                                                                                                              
`ACCCTATAGACGTAGCTGTGAGTACCAACCGAATAGCAATCCTTAAGTACGCCGTTCCGTTCTCAGAAGAGTTACTCATCTGGTCCGATGAAGCACAATTCGTCCTGACTGCCTCGGGTACTCTCACATCTAAGTCGGTTGAGTTGAACCTAACGACCCAGTTTGACGTACAGGACCGAGCGAGACCTTTTGGGATTGGGCGTAATGTCTACTTTGCTAGTCCGAGGTCCAGCTTCACGTCCATCCACAGGTACTACGCTGTGCAGGATGTCAGTTCCGTTAAGAATGCTGAGGACATT‑ACATCACACGTTCCTAACTACATCCCTAATGGTGTGTTCAGTATTTGCGGAAGTGGTACGGAAAACTTCTGTTCGGTACTATCTCACGGGGACCCTAGTAAAATCTTCATGTACAAATTCCTGTACCTGAACGAAGAGTTAAGGCAACAGTCGTGGTCTCATTGGGACTTTGGGGAAAACGTACAGGTTCTAGCTTGTCAGAGTATCAGCTCAGATATGTATGTGATTCTTCGCAATGAGTTCAATACGTTCCTAGCTAGAATCTCTTTCACTAAGAACGCCATTGACTTACAGGGAGAAC  >  NC_001604.1/26007‑26606`

  

Alignment Legend

Aligned base mismatch/match (shaded by quality score): `ATCG/ATCG` `< 3 ≤` `ATCG/ATCG` `< 11 ≤` `ATCG/ATCG` `< 23 ≤` `ATCG/ATCG` `< 35 ≤` `ATCG/ATCG` `< 38 ≤` `ATCG/ATCG`

Unaligned base: `atcg`    Masked matching base: `atcg`    Alignment gap: `‑`    Deleted base: `‑`
